# The Destructive Effects of Recreational Off-Highway Vehicles on Farmer Well-Being: Mental Health Consequences Outweigh Economic Losses

**DOI:** 10.3390/ijerph20054117

**Published:** 2023-02-25

**Authors:** Moshe Gish, Aya Shalmon, Ofira Ayalon

**Affiliations:** Department of Natural Resources and Environmental Management, University of Haifa, Haifa 3498838, Israel

**Keywords:** ATVs, farmers’ mental health, farmers’ well-being, farm stress, off highway vehicles, psychological well-being

## Abstract

In many countries, the use of recreational off-highway vehicles (ROVs) occasionally spills over into agricultural lands. The conflict between ROV users and farmers is escalating due to the growing popularity of ROVs. Determined action of authorities for mitigating the phenomenon may necessitate an understanding of the nature and extent of the actual damage caused by ROVs. However, it is currently unknown how ROVs harm agriculture and what is their main deleterious effect on farmers. We tested our hypothesis that economic costs are the leading reason for farmer distress using in-depth interviews with 46 Israeli farmers that are affected by ROVs. We found that contrary to our hypothesis, economic costs were low and negligible, despite high levels of anger, distress, or hopelessness expressed by almost all farmers. The main reason for outrage and frustration was the emotional impact of ROV activity on the farmers. Therefore, measuring the effects of ROVs on agriculture in terms of economic losses will probably be ineffective in convincing policymakers to act against the reckless use of ROVs in agricultural lands. On the other hand, conveying the emotional implications for the farmers can potentially promote change if accompanied by explanations on the importance of caring for the mental health and well-being of a sector that already suffers from levels of stress and mental health problems that are among the highest of any other industry in the world.

## 1. Introduction

In recent decades, the use of recreational off-highway vehicles (ROVs, including all-terrain vehicles (ATV), 4 × 4 trucks, dirt bikes, and other motor vehicles designed for off-road recreational use) has become one of the most popular forms of recreation in the USA [1,2] and in other countries [3]. The global market of ROVs is expanding exponentially and is estimated to grow from USD 15.7 billion (in 2022) to 54.4 billion in 2032 [4].

While ROV use in open areas provides pleasure, excitement, and social benefits to users [5,6], it often causes harm to the environment, especially where regulation and enforcement are lacking. ROV-induced damage in natural environments is well documented [7,8,9], especially in habitats that have low productivity, resistance, and resilience, such as boreal and arid ecosystems [10,11,12,13,14,15], dunes [16,17,18,19], and sandy beaches [20,21,22]. ROVs that drive in natural areas may negatively impact plants [11,17,19], animals [23,24,25,26,27,28], and soil [11,14,19,29,30,31] and can even facilitate the spread of alien species [32,33].

ROV users do not necessarily limit their activity to natural or unoccupied open areas. In many places, some ROV users enter agricultural fields, where they may cause damage to the crop, soil, field drainage, and irrigation systems. In some countries where the rise in ROV popularity has exacerbated the problem in agricultural lands, farmers occasionally express their outrage and distress in the public media (e.g., England [34], USA [35], Canada [36,37]). In Israel, ROV activity in agricultural areas has increased dramatically in recent years to the point that it has become a significant nuisance for many farmers throughout the country (A.S., Personal Communication with multiple farmers and several farmer organizations). Therefore, Israel—the setting for this study—is an excellent location for studying ROV effects on agriculture.

The high levels of anger and frustration typically expressed by farmers that are affected by careless ROV driving indicate that external stressors induced by ROV users affect the farmers’ mental well-being.

Many external factors are known to influence the mental health of farmers [38,39,40]. Among these factors, financial issues [41,42] are typically considered to be the most dominant [43,44], and consequently, economic stress has become the focus of most international initiatives for promoting farmer well-being [44]. However, if economic costs are indeed so significant, it is unclear why the demand of farmers in Israel (and possibly in other countries as well) for stricter legislation and law enforcement has so far fallen on deaf ears. It is possible that due to an absence of solid data on the consequences of ROV activity, farmers and farmer organizations have been unsuccessful in convincing policy makers that ROVs cause meaningful damage to them. Alternatively, farmer distress may be driven not by economic losses but by other overseen consequences of ROV riding that are more difficult to quantify and that should be brought forward in order to encourage authorities to take meaningful action. To the best of our knowledge, no study has ever attempted to identify the mechanism of the deleterious effects of ROVs on agriculture, presumably since only in recent years have ROVs emerged as a major nuisance to farmers. The goal of our study was, therefore, to fill the apparent gap between farmers and policy makers with insights into the true nature of ROV impacts on agriculture. Following the conventions in farmer well-being literature, we hypothesized that the economic stress imposed on the farmers by ROV users is the lead cause for their aggravation and distress.

## 2. Materials and Methods

The study was conducted in Israel from the beginning of winter 2020 until the end of spring 2021. A total of 46 farmers (45 men, one woman) who cultivate crops in the northern and central parts of the country were interviewed (the southern part of the country is a desert with sparse population and little agriculture, and therefore was not included in this study). Farmers were recruited through advertisements in social media and farming journals, asking those that have been affected by ROVs to take part in a study. This recruitment method created a self-selection bias, which made this study unrepresentative of the broader farmer population, as only farmers that have experienced ROV damage were included in the study. However, this was not a concern for our study, as our goal was not to provide a representative analysis of the farmer population but rather to compare the contributions of different factors that generate farmer distress. The study was approved by the ethics committee of the University of Haifa (protocol code 446/20; date of approval: 20 October 2020).

The farmers that contacted the researchers were initially given a link to an online survey that collected map-based data on spatial attributes of the phenomenon. The spatial analysis of ROV activity will be presented elsewhere. Afterward, the farmers were interviewed over the phone (38 farmers) or in person at the farmers’ fields (8 farmers). We composed the questionnaire that guided the in-depth interviews (Table 1) after preliminary discussions with three farmers, who also provided us with constructive feedback during the process of designing the questionnaire. When answering our questions, farmers were asked to refer to the last five years. We emphasized to the participants that their personal information would be kept confidential. This was important since many were initially hesitant to cooperate for fear of revenge by the ROV users. In these open-ended interviews (hereafter referred to as the “main survey”), almost all farmers were reluctant to provide financial information on the economic implications of ROV damage to their business (Table 1 question 9). Therefore, we contacted the farmers again several months later and asked them to rank their subjective feelings about the phenomenon’s economic and emotional effects on them (hereafter referred to as the “complementary survey”; Table 1 questions 15–17).

We used IBM SPSS Statistics (Version 27) to perform statistical analyses on the data, including Spearman’s rank correlation, McNemar’s test, and paired samples *t*-test. All averages are given with ±standard error of the mean (SEM).

## 3. Results

**Figure 1:**
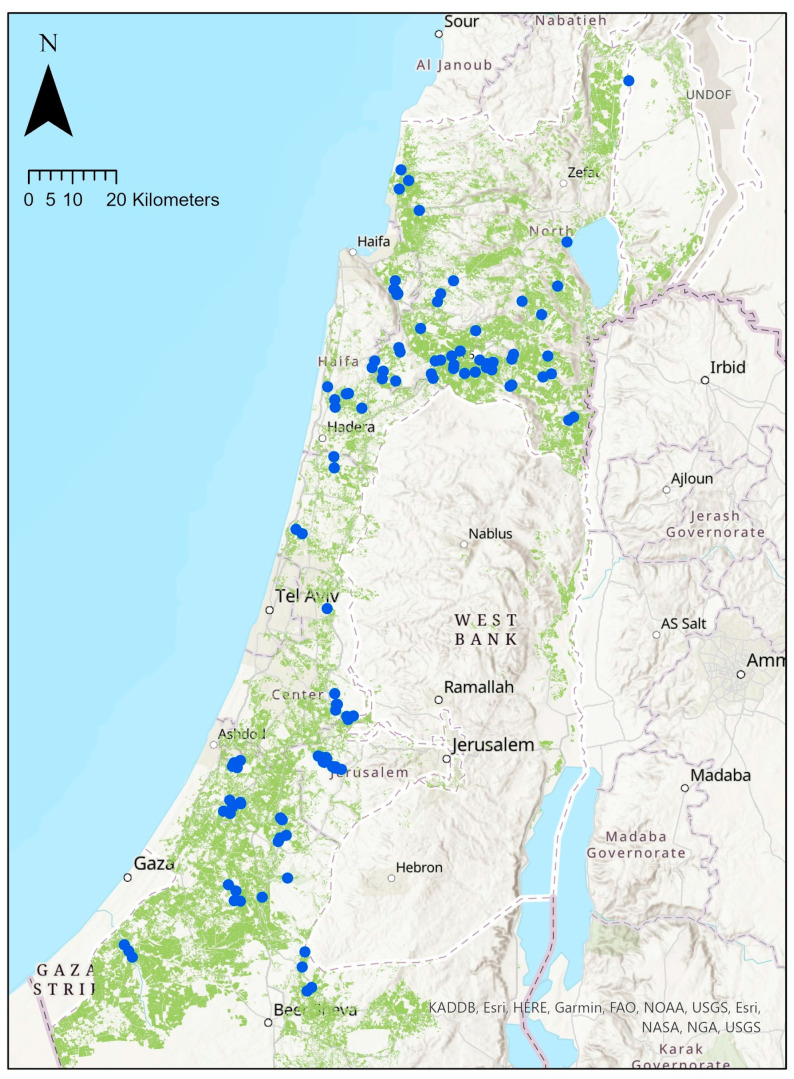
A map of northern and central Israel, with geographical locations (blue marks) of 119 reports on ROV activity events within agricultural fields, provided by the 46 farmers that participated in this study. Green areas represent agricultural fields of vegetables, field crops, and plantations.

The farms that were included in this study were distributed throughout northern and central Israel (Figure 1). Not all farmers gave answers to all the questions they were asked. The data presented here, therefore, refer to the answers that were received for each question. Wherever results are given as percentages of farmers, if not all farmers answered that question, exact numbers are also given in parentheses. The 46 farmers that were included in the study together hold a total of 45,100 hectares, which form 18.8% of all cropland areas in northern and central Israel [45]. Average farmer seniority was 26.8 ± 1.6 years in agriculture (range: 7–60; median: 27). Farm sizes and data on ROV events are given in Table 2.

While we did not aim to study the ROV riders themselves, we asked the farmers about their subjective opinions regarding the identity and motivations of the riders who entered their fields. Recreational riding was the most common purpose cited, mentioned as the sole motivation by 70% of farmers and as one of several motivations by 20% of farmers. A majority of farmers (79%, 34/43) indicated that they believed the riders were primarily local, hailing from nearby towns and villages.

All farmers but one reported that ROV activity in their fields has intensified in recent years. Most farmers (56%, 18/32) said that damage in their fields started within the past five years, 34% (11/32) said it began at least 10 years ago, and 9% (3/32) began noticing ROV activity at least 20 years ago. Most farmers (86%, 32/37) also said that ROV damage in their fields occurs mostly on weekends.

In general, the bigger a farm is, the smaller the percentage of the farm that has been damaged by ROVs (Spearman’s rho = −0.641, *p* < 0.001), and the smaller the number of damage events per hectare (Spearman’s rho = −0.608, *p* < 0.001) (Table 2).

Damage to crop plants was the main physical effect of ROVs, according to 96% of the farmers. Damage to irrigation systems was reported by 35% of the farmers. Wheel tracks that create obstacles for harvesting machines were reported by 15% of farmers, and tracks that damage field drainage were reported by 17%. Direct disturbance (when ROVs obstruct workers and machinery) was reported by 11%, but this was only reported for small- and medium-sized farms.

In the main survey (Table 1, questions 1–14), 80% of farmers said ROV activity makes them feel insulted, stressed, aggravated, or outraged [if farmers that gave an answer of eight or higher to the question about emotional effects in the complementary survey (Table 1, question 16) are included in this group, this figure goes up to 91%]. Most farmers (70%) expressed anger or disappointment toward the authorities (legislators, law enforcement agencies, courts) for their incompetence. Some farmers (17%) admitted that they had taken violent or illegal actions in the past (e.g., laying tire deflation devices, damaging the ROV with a crowbar), wish they could do so in the future or fear they eventually will. For example:

“Only a rifle and bullets will solve the problem”… “I would have killed him, but he stepped on the gas pedal and escaped”…. “we have violent incidents all the time”.

Farmer 1

“My dream is a drone with an M72 LAW anti-armor weapon on it”.

Farmer 2

“I do not go near them, because if I do, I will pull out a gun and shoot them”.

Farmer 3

Although the survey contained questions about damage type, extent, and frequency (Table 1), the evaluation of economic costs according to the farmers’ answers was challenging. In addition to not wanting to discuss their finances, many farmers said it is difficult for them to translate ROV damage into lost income since the damage is not always immediate or easily detected, and the extent and the ultimate consequences of ROV impact can have high spatial and temporal variation. Therefore, we used the average percentage of the field that was damaged every year in the past five years (Table 2) as a proxy for economic damage. Negligible damage to the field was reported by 26% of farmers, who explicitly said that there was minimal or no economic cost of ROV damage in their field (Table 1 question 9). Some provided only a vague reference to costs, but no farmer clearly stated that ROVs cause economically significant damage to their farm.

**Figure 2:**
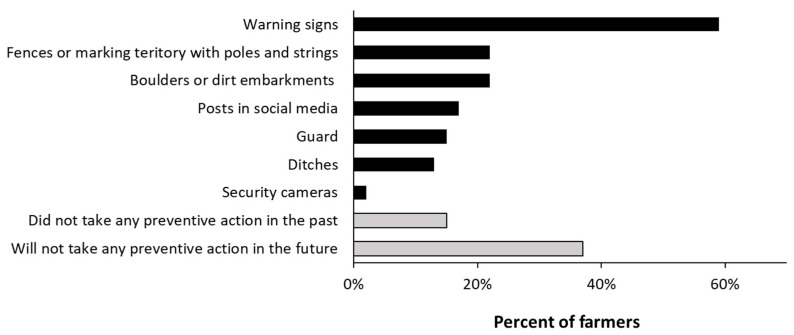
Actions farmers have taken in the past five years (blue bars) to reduce ROV damage in their fields.

Many farmers attempted to combat ROVs using different methods in the past five years (Figure 2).

The farmers found that some methods are more effective than others: closing roads with gates is effective in reducing ROV activity, but it might interfere with the activities of other farmers and tourists. Boulders were effective only against ROVs with more than 2 wheels. Signs, ditches, and dirt embarkments were considered completely useless against ROVs by 59% of farmers; signs tend to be ignored or stolen, and ditches and dirt embarkments actually attract thrill-seeking ROV riders instead of keeping them away.

From a future perspective, farmers that have used preventative measures against ROVs in the past five years tended to say they will not use preventive measures in the future (McNemar’s test, *p* = 0.013).

Similarly, farmers that have reported ROV damage events to the police in the past typically did not think they would report these events to the police in the future (McNemar’s test, *p* < 0.001).

A quarter (26%) of the farmers said that they do not leave their homes on weekends in order not to witness the ROVs that are driving in their fields. Statements indicative of hopelessness and despair were common. For example:

“I will not take anymore actions against it. We are hopeless. We do not know what to do… Some farmers will eventually lose their sanity”.

Farmer 4

“We have been fighting a losing battle for many years”.

Farmer 5

“We have surrendered. It is not easy to admit it. It is what it is”.

Farmer 6

In the complementary survey (Table 1, questions 15–17), 36 out of 46 farmers (78%) agreed to answer the three supplementary questions about how ROVs affect them economically and emotionally. The emotional impact on the farmers was very high (9.11 ± 0.23 on a scale of 1–10), and it was significantly higher than the economic impact (5.08 ± 0.42; paired samples *t*-test, *p* < 0.001). The average score given by the farmers in response to the question about their motivation to continue working in agriculture (Table 1, question 17) was 2.61 ± 0.43. Only six farmers answered the motivation question with a score that was higher than three. Out of the 30 farmers that gave a score of three or less (low probability that ROVs will negatively affect their motivation), 29 also reported a negative emotional effect higher than eight.

## 4. Discussion

The phenomenon of ROV damage in agricultural fields in Israel has been on the rise, especially in recent years. This trend was almost unanimously reported by the farmers who participated in this study. However, as we did not aim to quantify the intensity of the phenomenon throughout the country, we can only draw conclusions on the characteristics of damage when it occurs. Nevertheless, even though all farmers that were recruited were ones that have personally experienced the phenomenon, we can safely conclude that the conflict between farmers and ROV users has been escalating in affected agricultural areas.

The farmers that are most affected are those with smaller fields (Table 2), likely due to an edge effect (in a large field, a bigger proportion of the area is less accessible to ROVs). Therefore, per unit of area, the effect of ROVs is more meaningful for farmers with small farms. The fact that direct interference with farm work was only reported in small and medium farms also supports the conclusion that ROV damage may be greater in smaller farms.

The farmers that participated in this study overwhelmingly expressed high levels of stress, aggravation, and outrage caused by inconsiderate and irresponsible ROV riding in their fields. The fact that some of them have committed or said they might commit violent or illegal actions against ROV riders demonstrates the high levels of aggravation and distress they are subjected to. This finding aligns with the high levels of distress and aggravation expressed by farmers over the media in various countries, which have led us to our original hypothesis that it is the economic burden that exasperates the farmers. We were, therefore, somewhat surprised to find that the emotional effects of the phenomenon overshadow the economic costs to the farmers.

The fact that a quarter of the farmers said that the economic costs are negligible and none of the farmers reported meaningful losses supports the conclusion that physical damage is usually small. Additionally, even though the farmers’ responses to the question “…how significant for you is the economic damage caused by ROV activity…” (Table 1 question 15) were highly subjective, the average rating of the economic damage was almost half of the average rating of the emotional damage. It is, therefore, most likely that the primary factor driving farmer outrage and distress is the emotional outcome of ROV activity in their fields rather than economic losses.

Extreme stress and aggravation, which are not coupled with measurable economic losses, may not be sufficient to motivate authorities to act firmly against ROV riders. This speculation is backed by an interesting fact mentioned by some of the farmers: when they manage to catch or document an ROV user driving in their fields and try to register a complaint with the police, they are required to provide an estimate of the cost of the damage caused by that specific rider. Usually, the economic damage for that single event is so small that the complaint is not even registered. Therefore, in places where farmers’ mental health and well-being are not yet recognized as important issues that deserve the attention of the authorities, the fact that the economic implications of the phenomenon are often relatively small may impede an effective solution to the problem. In such cases, raising awareness among policymakers of the importance of farmer stress and well-being and the profound effects of ROVs on farmers’ emotions may encourage them to take effective action against the phenomenon.

In addition to distress and aggravation, the farmers that participated in this study showed signs of growing despair. There are several indicators that support this conclusion: (1) Even though most of the farmers have attempted to use various preventive measures in the past, many do not plan to take any action in the future in an attempt to keep ROV riders out of their fields (Figure 2). Some of these farmers may have learned that such efforts are futile, and some may simply be tired of fighting. (2) Farmers that have reported to the police in the past tend not to see themselves attempting to register complaints in the future. (3) A quarter of the farmers said they stay at home on weekends in order to avoid aggravation. The fact that ROVs are active mostly during the weekend indicates that the activity is mostly recreational in nature, and it is likely that seeing people taking pleasure in sabotaging their work is unbearable for those farmers.

Interestingly, even though the feeling of hopelessness seems to be growing among farmers, their motivation to continue working in agriculture is still very high. It is noteworthy that almost all farmers that gave a high rating to the negative emotional effects of ROVs on them gave a low rating to the probability that they will quit working in agriculture. Therefore, it seems that until ROVs come under control, they will continue tormenting the farmers, taking a toll on their mental health.

In recent years, studies on farmer mental health have raised concerns over the exceptionally high levels of stress, anxiety, and depression among farmers throughout the world [38,39,46,47]. In a recent US survey, most farmers said mental health is a very important issue to them and their families [43]. Several government bodies and other organizations have emphasized the importance of recognizing and dealing with farmer stress (e.g., [48,49,50]), and numerous university extensions provide resources for farm stress management (e.g., [51,52,53,54]). The demanding and sometimes unrewarding work under challenging conditions makes farmers exceptionally susceptible to mental health problems [38]. One of the outcomes, for example, is that farmers in many countries have suicide rates that are among the highest of any other industry [39,55,56,57,58]. The elevated levels of stress, anxiety, and mental problems have been shown to degrade the well-being of farmers and their families [59,60]. A manifold of external influences may increase farmer stress levels, including weather uncertainty, crop failure, financial issues, farm crime, social isolation, and government policy and bureaucracy [38,39,40]. In a past study on environmental stressors, the feeling of being unsupported and not valued by the government and the public was a common stressor among Australian farmers [40]. A similar sentiment stood out in the interviews we conducted, as most farmers expressed anger or disappointment over the carelessness and incompetence of the authorities.

Promoting mental health and well-being is part of the United Nation’s Sustainable Development Goal (SDG) 3.4 [61]. Mitigating farmer stress, thus improving the well-being of agricultural communities, should therefore be a priority social issue, especially in the face of climate change, which may exacerbate farmer mental health problems throughout the world [62,63,64,65,66]. Moreover, the fact that the phenomenon of ROV damage to agricultural fields is on the rise and the expectancy that ROVs will increasingly become more popular in the future emphasizes the urgency of addressing this issue.

Some of the environmental stressors that affect farmers are difficult, complicated, or impossible to resolve (e.g., weather uncertainty, physically demanding work and long working hours, social isolation, financial pressure, crime, etc.). However, mitigation of ROV-induced farmer stress in affected areas may be achievable by improving legislation and enforcement, as well as implementing taxes on ROVs that reflect their environmental impact. The benefit–cost ratio of these actions may be very high if one considers farmer mental health to be an important issue [44]. However, for this to happen, decision makers need to be convinced that the problem is meaningful, even if it cannot be directly measured in terms of money.

Our study demonstrates what is often ignored by researchers and governmental institutions: the farmer’s well-being is a multidimensional concept that is not necessarily dominated by their economic well-being [44]. We contend that studies such as this one and others that may follow could facilitate a much-needed change in the way societies treat their farming communities by shedding light on the overlooked consequences of recreational activity that, by definition, should involve nothing but fun and enjoyment for everyone.

## 5. Conclusions

This study provides insight into the phenomenon of ROV damage in agricultural fields and how it impacts farmers. The results indicate that the conflict between farmers and ROV users in our study area is escalating in affected agricultural areas, with smaller farms being the most affected. We also found that farmers experience high levels of emotional stress, aggravation, and the outrage caused by ROV activity in their fields. The study suggests that these emotional effects are far more significant than the economic costs, with many farmers finding it difficult to translate ROV damage into lost income. The study highlights the need for authorities to recognize the deleterious impact of uncontrolled ROV activity on farmers’ mental health and well-being and to take action to address the problem, even if it does not necessarily translate into quantifiable economic losses.

## Figures and Tables

**Table 1:** Questions that guided the in-depth interviews with the farmers.

Number	Question
Main survey	
1	Where are your fields located and what is their total size?
2	How long have you been a farmer?
3	How many ROV events do you experience in your fields every year, on average?
4	What is your assessment for the average size of the area in your fields that is damaged annually (in hectares)?
5	How many years ago did ROVs start affecting your fields? (1–20 years ago)
6	Do you think there is a trend in recent years in the severity of the phenomenon?
7	Are ROVs active in your fields mostly during the week or over the weekend?
8	What type of physical damage do ROVs cause in your fields?
9	What is the average yearly economic cost of ROV damage to your business? (setup and maintenance of barriers and signs, repairs, crop loss, etc.)
10	Have you ever tried placing physical obstacles or warning signs around your fields to prevent ROVs from entering? If so, what have you tried, and how effective was it?
11	Do you think you will use physical obstacles or warning signs in the future?
12	Did you report the events to the police in the past?
13	Do you think you will report events to the police in the future?
14	Can you elaborate on the effects of ROVs on you as a farmer and on your business?
Complementary survey	
15 *	All in all, when it comes to your net profit, how significant for you is the economic damage caused by ROV activity in your fields (on a scale of 1–10)?
16 *	How significant for you are the negative emotional effects of ROV activity in your fields (on a scale of 1–10)?
17 *	To what extent do you think ROV activity may negatively affect your motivation to continue working in agriculture in the future (on a scale of 1–10)?

* Questions asked in the complementary survey conducted several months after the completion of the main survey. Unless stated otherwise, all questions refer to the past five years.

**Table 2:** Sizes of the farms included in the study and ROV events that took place in the past five years. n = number of farmers in the category that answered the question.

Farm Size (Hectares)	Number of Farms in the Category	Average Number of ROV Events per Year	Average Number of ROV Events per Year per 100 Hectares	Average Percentage of the Farm That Has Been Damaged by ROVs Every Year
Small (40–450)	20	78 ± 24 (n = 11) (range: 2–300; median: 25)	24.4 ± 9.9 (range: 1.6–100; median: 8.9)	5.3 ± 1 (n = 13) (range: 0.01–10; median: 5)
Medium (500–1000)	13	38 ± 16.6 (n = 12) (range: 1–200; median: 15)	6.3 ± 2.5 (range: 0.1–28.6; median: 2.5)	2.6 ± 0.6 (n = 6) (range: 0.5–5; median: 2.5)
Large (1150–2000)	7	65.8 ± 37.6 (n = 6) (range: 5–250; median: 40)	5 ± 3.4 (range: 0.3–21.7; median: 2.3)	1.5 ± 1.2 (n = 4) (range: 0.02–5; median: 0.04)
Very large (2200–5000)	6	52 ± 25.4 (n = 5) (range: 10–150; median: 30)	1.9 ± 1.2 (range: 0.4–6.8; median: 1)	0.6 ± 0.4 (n = 3) (range: 0.03–1.5; median: 0.04)
Number of farmers that did not answer question		12	12	20

## Data Availability

All relevant data are presented within the paper. Full interviews are available on request from the corresponding author. The data are not publicly available due to the confidentiality of participants.
